# An Address

**Published:** 1865-07

**Authors:** G. L. Paine


### AN ADDRESS.

BY DR. G. L. PAINE.

**Read before the Mad River Valley Dental Association.**

*Retrospective, Prospective and Descriptive.*

GENTLEMEN :—

Allow me to congratulate you, that you have again been permitted to meet under such favorable auspices. The Storm cloud of War, which hung so portentously dark over our land for four long years, causing unutterable woe, anguish and suffering, has passed away, followed by the sunshine of Peace! No longer shall we hear the clash of resounding arms—the roar of hostile cannon—no longer see the heavens lightened up with the flames of burning cities, mills, factories and barns—no longer shall the soil of this goodly land be soaked with the blood of our countrymen, slaughtered by a barbarous foe. No longer shall our gallant patriots famish and die, in Libby and Andersonville prison pens. The last shot has been fired,—the last bugle blast blown—the last signal gun heard—the last prisoner taken, and thank GOD and Lincoln, the last slave made free. The last surrender has been made—the last Rebellion in America and the greatest in history, has been crushed to atoms, by the prowess of our gallant soldiers, directed by the genius of a Grant, and his able co-adjutors, Sherman, Sheridian and Thomas. The arch traitor Davis—late President of the bogus confederacy, and the chieftain of the rebel forces has been captured and now a prisoner, pines in a felon's cell, indicted by his fellow countrymen, for the high crime of Treason against the best government of Earth.

Peace, giving liberty to the captive and freedom to the slave, follows in the wake of horrid war.

But one flag floats in the breeze and that is the time honored "Star Spangled Banner"—but one navy floats in our waters, and but one government exists, that founded by our fathers, and now re-established by four years of hard fighting by their gallent sons. I notice that one of our number—one of the original members of this Society is not here, but absent, not as some others are, from sordid motives but absent because dead, a martyr to liberty. He died the death of a hero, pouring out his young blood on the rocks of Gettysburgh while gallantly urging on his braves, may the rains of Heaven fall gently on the sod that covers the bosom of the gallant Major, may his memory be cherished by us. He with scores of thousands stood like a wall of fire between us and our enemies. Keeping them at bay, else, perhaps we had not been here to-day to utter this feeble eulogy to his name and deeds.

We are signally blessed in having been permitted to see this glorious day, glorious because the war is ended, law and order reign, right and truth are established and slavery forever abolished. Now that Peace has returned to us with her spring face and the business of the country likely to be established on a firm basis and a heavy demand for professional services at our hands in new prospect, after having laid aside our sword and musket let us take up and use effectively the excavator, chisel and mallet, never to be laid aside, till we are summoned to cross over the dark Jordon of Death—there to join in the spirit land, the great and good of our profession who have gone before.

Let us be ambitious to attain skill and proficiency in all our operations, deciding to do *well*, whatever we do, though that be but little; rather than trying to do a great deal, when of necessity much of that may be imperfectly done, very likely to our lasting injury and the disgrace of the profession. Even if we are governed by the sordid motives of gain, and I greatly fear that, that is the crying sin of many in the profession, no investment pays so large a dividend, as an honest and determined effort *to do our best at whatever cost.* It is as true in Dentistry as in morals—" As ye sow, so shall ye reap." Cultivate your conscience, daily, hourly—determined to be known and distinguished for your scrupulous adherence to truth and honesty in all your relations and dealings with the world, with the profession and especially and unfailingly with your patients, whose confidence should never be violated, nor their trust betrayed. It is a great calamity—a standing reproach—a foul blot upon our noble profession, that in its wake follow so many, lying, scoundrelly, rascally fellows, who grow sleek and fat by falsehood, misrepresentation and knavery. They axaggerate, they magnify and amplify, as to the amount of their business the quality of their work and its perfection and success. They agonize to become conspicuous, and sometimes resort to the holy sanctuary, there to beslaver the same with the slime of their hypocrisy. In their desperate and frantic efforts to obtain business, they visit every farm house in the by-ways and hedges, like their brethren the Jews, and thrust their attention upon all; and *beg, tease, coax and flatter*, until the victim unable longer to resist the tremendous pressure, yields and is speedily victimized of his teeth and money, by these dental Boa Constrictors. They are quite willing in the event of any impecuniosity on the part of their victims, to exchange their wares for any sort of produce, such as hay, corn, oats, butter, eggs, chickens, coal or cordwood, potatoes or petroleum. Such gentlemen, are some of the characteristics of many creatures, who assume to be dentists ; who repudiate dental colleges, dental societies, and all dental fellowship or association. They are dental Ishmailites, in arms against the whole profession. Enveloped in the lion's skin, they appear great only in their dens; abroad among their betters, who practice their profession legitimately, the lion's skin is no protection, as the asses ears are sure to stick out. Hence, their faces are never seen in dental meetings, fearing forsooth, some duty will be imposed to which they would be unequal and their poverty and nakedness be thus exposed, their prestige would vanish, like the mists of the morning. Hence they prefer to stay at home, in order to pursue their calling; the only one for which they have any fitness; *making money;* the chief object of their being—the only article in their creed—"their Shorter Catechism" and their "Confession of Faith." These dental colporteurs are of course birds of passage; migratory in their habits, soon playing out because they build their houses on sandy foundations.

We have held up the glass to and photographed these fellows that they may see themselves as other see them. Hoping gentlemen, that we may have a pleasant and profitable session; that we may all be thoroughly alive to every good word and work, that our socieiy may grow both in number and strength, till a moral influence shall go out from it, that may be felt throughout the whole valley; that we may both here and elsewhere deport ourselves like men, ever discharging our duties with an enlightened conscience and "with an eye single to the honor and glory of GOD" is our desire; then all will be well.

